# Natural course of cerebral and spinal cavernous malformations: a complete ten-year follow-up study

**DOI:** 10.1038/s41598-023-42594-0

**Published:** 2023-09-19

**Authors:** Alejandro N. Santos, Laurèl Rauschenbach, Hanah H. Gull, Angelina Olbrich, Thiemo F. Dinger, Marvin Darkwah Oppong, Christoph Rieß, Bixia Chen, Annika Lenkeit, Börge Schmidt, Yan Li, Ramazan Jabbarli, Karsten H. Wrede, Adrian Siegel, Ulrich Sure, Philipp Dammann

**Affiliations:** 1 Department of Neurosurgery and Spine Surgery, University Hospital Essen, Hufelandstrasse 55, 45147 Essen, Germany; 2 Institute for Medical Informatics, Biometry and Epidemiology, University Hospital of Essen, Essen, Germany; 3 Institute of Diagnostic and Interventional Radiology and Neuroradiology, University Hospital Essen, Essen, Germany; 4 Department of Neurology, University of Zurich, Zurich, Switzerland; 5 Center for Translational Neuroscience and Behavioral Science (C-TNBS), University of Duisburg-Essen, Essen, Germany

**Keywords:** Risk factors, Cerebrovascular disorders, Neurovascular disorders, Spinal cord diseases

## Abstract

Knowledge of the bleeding risk and the long-term outcome of conservatively treated patients with cavernous malformations (CM) is poor. In this work, we studied the occurrence of CM-associated hemorrhage over a 10-year period and investigated risk factors for bleeding. Our institutional database was screened for patients with cerebral (CCM) or intramedullary spinal cord (ISCM) CM admitted between 2003 and 2021. Patients who underwent surgery and patients without completed follow-up were excluded. Analyses were performed to identify risk factors and to determine the cumulative risk for hemorrhage. A total of 91 CM patients were included. Adjusted multivariate logistic regression analysis identified bleeding at diagnosis (*p* = 0.039) and CM localization to the spine (*p* = 0.010) as predictors for (re)hemorrhage. Both risk factors remained independent predictors through Cox regression analysis (*p* = 0.049; *p* = 0.016). The cumulative 10-year risk of bleeding was 30% for the whole cohort, 39% for patients with bleeding at diagnosis and 67% for ISCM. During an untreated 10-year follow-up, the probability of hemorrhage increased over time, especially in cases with bleeding at presentation and spinal cord localization. The intensity of such increase may decline throughout time but remains considerably high. These findings may indicate a rather aggressive course in patients with ISCM and may endorse early surgical treatment.

## Introduction

Cavernous malformations (CM) of the central nervous system (CNS) are considered as one of the most frequent neurovascular malformations^1–3^ and a major cause of intracerebral (ICH) or intramedullary (IMH) hemorrhage^1,4–6^. Although they generally tend to have a benign natural history (usually requiring only clinical and radiological follow-up)^1,4,6,7^, CM-related ICH/IMH can lead to significant morbidity with seizures and/or severe focal neurological deficits^1,4,7–13^. A considerable number of observational and population-based studies assessed the natural course of the disease and risk factors increasing the bleeding risk (e.g. lesion localization, previous hemorrhage, presence of developmental venous anomaly (DVA))^1,5,14–19^. These studies estimate a cumulative risk of ≈20% over a 5-years’ timeline for patients with cerebral cavernous malformations (CCM)^1,2,16,19^, as well as ≈40% for patients with an intramedullary spinal cavernous malformations (ISCM)^4,5,7^. Notably, such trials are usually limited to rather small cohorts, a majority of surgically treated cases, and short follow-up periods^1,4,5,8,16,17,19,20^. Moreover, most of these studies segregate this disease according to the localization of the lesion (lesion located in the brain or spine), although it regards a disease that concerns the entire CNS. Consequently, information regarding the natural course of this disease in the entire CNS throughout a longer follow-up period could significantly impact current treatment approaches. In a previous paper from our group, Rauscher and colleagues studied CM patients for the first time over a ten-year period. In this study, the influence of modifiable vascular risk factors on bleeding frequency was investigated, but no association could be observed^21^. Thus, it remains unanswered whether other factors exist that influence bleeding behavior within 10 years. Based on the published cohort of Rauscher and colleagues, we therefore investigated other factors for their association with hemorrhage.

## Methods

### Data collection

Informed consent was obtained from all subjects involved in the study. The study was performed according to the STROBE protocol. This study was conducted at our tertiary university hospital, according to the principles of the Declaration of Helsinki and guidelines of an approving institutional review board, as well as ethics committee (Ethik-Kommission, Medizinische Fakultät der Universität Duisburg-Essen, Registration number 14-5751-BO and 19-8662-BO). All methods were performed in accordance with the relevant guidelines and regulations of the above-mentioned institutional review board. In this observational cohort study, data were collected ambispectively, with a large proportion of data collected prospectively and a small proportion collected retrospectively. Part of the data was taken from the data of Rauscher and colleagues^21^. Screening for more suitable patients, we investigated all consecutive CM patients admitted to our department between 2003 and 2021. Study inclusion required patients with complete available magnetic resonance imaging (MRI) dataset and baseline clinical characteristics such as age at diagnosis, sex, CM location, CM multiplicity, CM family history, presence of cavernoma-related epilepsy (CRE), presence of DVA, as well as a minimum follow-up of 120 months. Surgically treated patients during the follow-up time were excluded from the study. Surgical removal of a lesion in a patient with multiple CM did not lead to censoring and follow-up assessment continued for the remaining lesions. We defined familial disease according to standard definitions: genetically confirmed by testing and/or known affected relatives and/or absence of associated DVA^22,23^. Clinical presentation was assessed at diagnosis, after initial hemorrhage, and after recurrent hemorrhage. Diagnosis of CM, CM-related ICH/IMH, lesion localization, and presence of DVA were assessed through MRI findings and confirmed by independent neuro-radiologists. We determined mode of presentation of all patients using the following classification: symptomatic hemorrhage with focal neurological deficit, focal neurological deficit not associated to a bleeding, or asymptomatic with non-hemorrhagic event. Follow-up data was obtained through routine examinations in our specialized outpatient clinic. Primary endpoint of the study was the occurrence of ICH/IMH during the follow-up period according to reporting standards for CCM^9^ as follows: acute or subacute onset of neurological symptoms related to the anatomical region of the lesion accompanied with radiological evidence of acute bleeding of the CM on a recent MRI. The final decision (if an event was counted as symptomatic hemorrhage) was reviewed, discussed, and ultimately decided by AS, LR, YL and PD, based on MRI findings and patient/parents’ interview/examination in each case. To be noted, part of our data has been previously published in a study, investigating the relationship between modifiable vascular risk factors and hemorrhage in patients with a CM in the CNS over a complete 10-year follow-up period^21^.

### Statistical analysis

**Figure 1:**
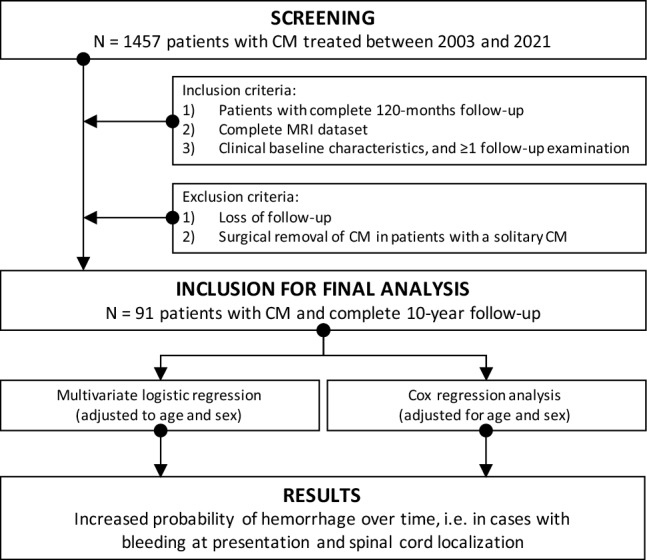
Design of the study. The figure illustrates the respective steps, states inclusion and exclusion criteria, and presents the main results of the study.

We used SPSS 27 (IBM, Armonk, NY, USA) for all statistical analyses. Associations of possible risk factors with hemorrhage during follow-up were assessed by calculating adjusted Odds Ratios (aOR) and 95% Confidence Intervals (95% CI) using a multivariate logistic regression model adjusted for age and sex. Identified risk factors (alpha-level < 0.05) were examined in a subsequent Cox regression analysis adjusted for age and sex to calculate adjusted Hazard Ratios (aHR) and 95% CI.^24^ Kaplan–Meier curves were obtained for the complete cohort and stratified by the implementation of statistically significant factors leading to CM-associated bleeding. Log-rank test was used to compare data of the Kaplan–Meier curves. Data were censored if patients experienced (re)bleeding. Results were considered statistically significant with an alpha-level of < 0.05. Selection and steps of our analysis are visualized in a flow diagram in Fig. 1.

## Results

### Characteristics

Ninety-one patients with CM of the CNS were included in our study. The mean age was 32.56 ± 14.59 years. A total of 61 patients (67%) were female. Twenty-seven patients (30%) revealed a lesion in the brainstem and 6 individuals (7%) in the spinal cord. Familiar history was diagnosed in 26 patients (29%). Forty-five patients (49.5%) presented with multiple CM. DVA was seen in 26 patients (29%). A total of 45 (53%) CCM as well as 4 (67%) ISCM patients suffered from hemorrhage at diagnosis. Twenty-seven patients (30%) suffered from (re)hemorrhage during the 120-month follow-up, and 5 (6%) of those patients presented with two bleeding events during follow-up. Detailed cohort characteristics are summarized in Table 1.

**Table 1:** Demographic, anatomic and clinical characteristics.

Characteristic	Frequency
Total number of patients with CM, n	91
Age, years, mean ± SD	32.56 ± 14.59
Female sex, n (%)	61 (67%)
Multiple cavernomas (≥ 2 CM), n (%)	45 (49.5%)
FD^a^, n (%)	26 (28.6%)
BSCM, n (%)	27 (29.7%)
ISCM	6 (6.6%)
Hemorrhage at presentation, n (%)	49 (53.8%)
CRE, n (%)	26 (28.6%)
Asymptomatic, n (%)	22 (24.2%)
DVA^b^, n (%)	26 (28.6%)
(Re)-hemorrhage during follow-up period, n (%)	27 (29.7%)
Second bleeding during follow-up period, n (%)	5 (5.5%)

*BSCM* brainstem cavernous malformation; *CM* cavernous malformation; *CRE* CM-related epilepsy; *DVA* developmental venous anomaly; *ISCM* intramedullary spinal cord CM; *FD* familial disease; *SD* standard deviation.

^a^12 patients missing.

^b^6 patients missing.

### Annual and cumulative 10-year risk of (re)hemorrhage

Kaplan–Meier curve analysis was implied to assess the risk of (re)hemorrhage during the 10-year follow-up period. We observed a total of 32 events occurring for 910 person-years (leading to 3.5 events per 100 person-years). The cumulative risk for hemorrhage within 10 years was 30% (95% CI = 21–40%) for the whole cohort, 39% (95% CI = 26–54%) for patients with bleeding at diagnosis, and 67% (95% CI = 24–94%) for patients with an ISCM. In addition, this risk was of 33% (95% CI = 17–54%) for patients with CM in the brainstem, 31% (95% CI = 16–51%) for patients with a familial CM disease and 0% (95% CI = 0–19%) for asymptomatic patients. The annual risk of (re)hemorrhage in the overall follow-up was 4% for the entire cohort, 5% for patients with bleeding at presentation, 4% for patients with a DVA, 7% for patients with a spinal cavernous malformation, 4% for patients with brainstem localization and 0% for asymptomatic patients. Data is provided in Table 2.

**Table 2:** Annual hemorrhage rates per patient with CM.

Parameter	Total person-years	No. of events	Annual bleeding rate (%)
All patients	910	32	3.5
Bleeding at diagnosis	490	22	4.5
DVA	260	10	3.8
BSCM	270	11	4.1
ISCM	60	4	6.7
FD	260	10	3.8
Asymptomatic	220	0	0

*BSCM* brainstem cavernous malformation; *CM* cavernous malformation; *DVA* developmental venous anomaly; *ISCM* intramedullary spinal cavernous malformation.

### Predictors of (re)hemorrhage

Known and potential risk factors for the occurrence of CM-associated hemorrhage were examined in a multivariate logistic regression model with respect to their influence on the risk of bleeding. Regression analysis adjusted for age and sex identified hemorrhage at diagnosis (aOR = 2.41 [95% CI = 1.05–5.54], *p* = 0.039), as well as localization of the lesion in the spinal cord (aOR = 4.20 [95% CI, 1.40–12.57], *p* = 0.010) as risk factors for the occurrence of (re)hemorrhage during the 10-year follow-up. Detailed data is provided in Table 3. Using collinear statistics, we additionally calculated the variance inflation factors (VIF) to quantify the severity of multicollinearity in a set of multiple regression variables. Notably, VIF values ranged between 1.021 and 1.151, indicating absence of multicollinearity.

**Table 3:** Multivariate logistic regression analysis adjusted for age and sex: cumulative 10-year (re)bleeding risk based on baseline characteristics.

Parameter	*p*-Value	aOR	95% CI
Bleeding at diagnosis	**0.039**	2.41	1.05–5.54
DVA	0.421	0.70	0.29–1.68
BSCM	0.599	1.25	0.55–2.83
ISCM	**0.010**	4.20	1.40–12.57
FD	0.372	1.47	0.63–3.44

*aOR* adjusted Odds Ratio; *BSCM* brainstem cavernous malformation; *DVA* developmental venous anomaly; *FD* familial disease; *ISCM* intramedullary spinal cavernous malformation.

Bold font indicates statistical significance.

**Figure 2:**
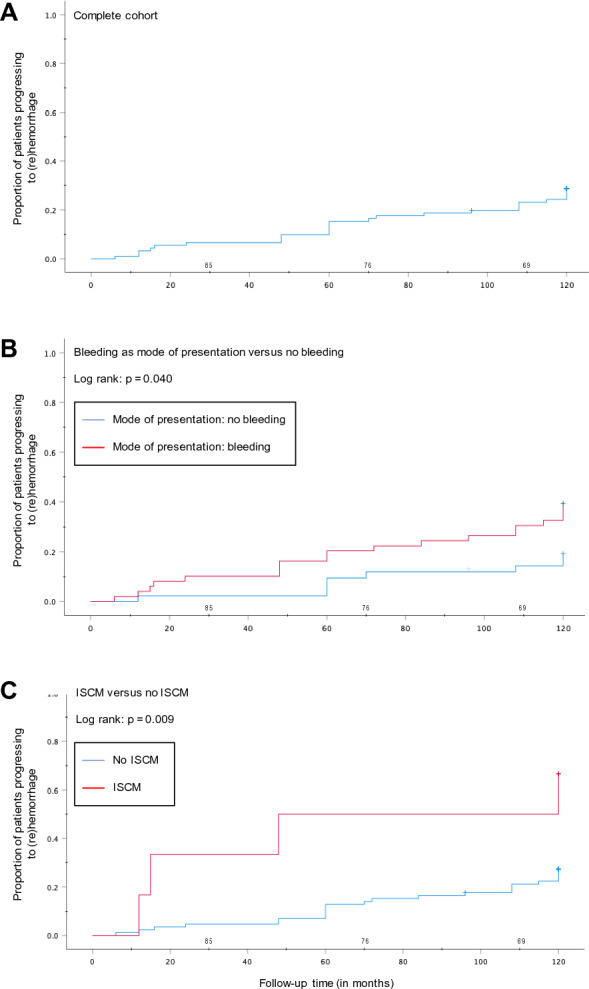
(**A**) Kaplan–Meier curves of the complete cohort, (**B**) stratified by bleeding as mode of presentation (hemorrhage as mode of presentation shown in red), and (**C**) stratified by CM localized in the spinal cord (lesion located in the spinal cord shown in red) over a 10-year follow-up. *CM* cavernous malformation; *ISCM* intramedullary spinal cord CM; *MOP* mode of presentation. *Note*: The numbers of patients at risk are shown on top of the x-axis of each graph. Log-rank test showed significant differences between groups in B and C.

In a next step, both risk factors from the logistic regression model were fed to a multivariate Cox regression analysis. Regression analysis adjusted to age and sex confirmed bleeding at diagnosis (aHR = 2.31 [95% CI = 1.00–5.35], *p* = 0.049) and ISCM (aHR = 3.91 [95% CI = 1.29–11.78], *p* = 0.016) as independent risk factors. Moreover, a Kaplan–Meier analysis was performed to visualize the risk of bleeding during the 10 years of follow-up. A log-rank test revealed significant differences between patients with or without a previous bleeding event (*p* = 0.040) and between patients with or without ISCM (*p* = 0.009). Data is illustrated in Fig. 2.

Finally, we repeated multivariate Cox regression analysis adjusted to age, sex, and hemorrhage at diagnosis. Notably, ISCM remained an independent predictor for the occurrence of (re)hemorrhage within the 10-year follow-up (aHR, 3.24 [95% CI = 1.11–9.45], *p* = 0.032). Data is illustrated in Table 4.

**Table 4:** Multivariate Cox regression analysis adjusted for age, sex, and hemorrhage at diagnosis.

Parameter	*p*-Value	aHR	95% CI
DVA	0.256	0.64	0.30–1.38
BSCM	0.672	0.83	0.35–1.97
ISCM	**0.032**	3.24	1.11–9.45
FD	0.811	1.06	0.64–1.77

*aHR* adjusted Hazard Ratio; *BSCM* brainstem cavernous malformation; *DVA* developmental venous anomaly; *FD* familial disease; *ISCM* intramedullary spinal cavernous malformation.

Bold font indicates statistical significance.

## Discussion

CM of the CNS is considered the second most frequent neurovascular malformation^1–3^ and a major cause of ICH^1,6^ and IMH^4,5^, which can potentially cause considerable morbidity^1,8–12^. Numerous studies exist, assessing their natural course and possible risk factors leading to ICH or IMH. Unfortunately, such studies are often associated with small cohorts, as well as a limited follow-up period^1,4,5,8,14–20^. The latter often occurs due to considerable amount of patients lost to follow-up up or surgical removal of their lesion^5,14,16,17,19^. To this extent, our study tried to analyze the natural course of conservatively treated patients with CM over an unprecedented long and complete follow-up period. Based on one of our previous studies examining the impact of vascular risk factors on bleeding risk in CM patients over 10 years, we assessed the cumulative incidence of hemorrhage and recurrent hemorrhage and examined possible risk factors for bleeding events^21^.

### Cumulative 10-year (re)hemorrhage rates during follow-up

The available literature mentions a cumulative risk of ≈20% over a 5-years’ timeline for patients with CCM^1,2,16,19^, as well as ≈40% for patients with ISCM^4,5,7^. Risk factors, such as spinal cord localization or ICH/IMH as mode of presentation increasing this risk are well known^1,4,5,20^. Our 10-year follow-up investigation showed a progressive increase of hemorrhage incidence throughout time, attaining almost a 30% chance of bleeding after 10 years, with an increase in risk from 20 to 30% after 5 years. Our study identified ICH as mode of presentation and spinal cord localization as risk factors for (re)hemorrhage. The latter has been assessed in a few number of studies, finding similar results^4,5^. Moreover, previous studies have assessed an annual risk of hemorrhage of ISCM between 1.7 and 10%.^4,5,7,25–28^. Such differences could be explained due to different elements such as divergent definitions of hemorrhage^9^, or baseline characteristics between studies^4,5,13,28^. Our study revealed a rate of 7%, which is within the range of the expected risk. Furthermore, we found no IMH during follow-up in asymptomatic patients. Previous series described similar findings.^4,26,29^.

A significant number of studies in the literature have well documented that hemorrhage at presentation is a strong predictor of recurrent bleeding after diagnosis^1,4,5,8,16,17,19,20^. Our study confirmed such findings. Our cumulative 10-year analysis revealed a progressively decreasing risk of hemorrhage after each year in the entire cohort, as well as a significant increase of this risk when only considering patients with ICH or IMH at presentation. Interestingly, our findings showed a rather high risk of (re)hemorrhage in patients with ISCM compared to those with CCM. This could indicate a more aggressive course in patients with ISCM.

### External validity

Compared to other studies regarding patients with CCM, our cohort seems to be representative in terms of patient characteristics. Compared to Horne et al. (the largest meta-analysis study assessing natural history) baseline characteristics were similar, with mean age of 33 vs 45 years, 30% vs 35% CCM in the brainstem, and 54% vs 51% presented with ICH at diagnosis. Our study shows a 100% completeness of the 10-year follow-up with no censoring due to loss of follow-up or surgical treatment during follow-up. It represents one of the few complete 10-year follow-up study to date and contributes novel and robust data. Future studies are needed to add data on this rare disease and to confirm our results.

### Limitations

Although being a prominent neurovascular disease, CCM only accounts for 10%– 15% of all intracranial vascular malformations^1,2^, and ISCM represents only ≈5% of all cavernous malformations, making CM of the CNS a rather rare vascular disease, which renders single-center studies with large cohorts strenuous. Our data was assessed from our tertiary referral center. This can lead to well-known information and selection biases. Moreover, data were collected ambispectively, with a large proportion of data collected prospectively and a small proportion collected retrospectively. This inevitably leads to a certain degree of information and selection bias. Finally, the rather small size of the subgroups in our cohort may have led to a known small sample bias. In particular, logistic regression analyses require a large number of patients and events, so the power of our results remains limited. Since the analyses in this work are based in part on published data, all limitations of the previous publication apply.

## Conclusions

During an untreated 10-year follow-up, the probability of (re)hemorrhage tends to increase over time, especially in cases with bleeding at presentation and spinal cord localization. The intensity of such increase may decline throughout time but remains considerably high. Our results suggest a more aggressive course in patients with ISCM, although the small number of cases significantly limits the robustness of the analyses. Future studies need to investigate this association, which may impact future treatment decisions for ISCM.

## Data Availability

The datasets generated during and/or analyzed during the current study are available from the corresponding author on reasonable request.

## Abbreviations

BSCM: Brainstem cavernous malformation
CCM: Cerebral cavernous malformation
CM: Cavernous malformation
CRE: Cavernoma-related epilepsy
DVA: Developmental venous anomaly
ICH: Intracerebral hemorrhage
IMH: Intramedullary hemorrhage
ISCM: Intramedullary spinal cavernous malformation

## References

[1] Horne MA, Flemming KD, Su IC (2016). Clinical course of untreated cerebral cavernous malformations: A meta-analysis of individual patient data. Lancet Neurol..

[2] Taslimi S, Modabbernia A, Amin-Hanjani S, Barker FG, Macdonald RL (2016). Natural history of cavernous malformation. Neurology.

[3] Robinson JR, Awad IA, Little JR (1991). Natural history of the cavernous angioma. J. Neurosurg..

[4] Goyal A, Rinaldo L, Alkhataybeh R (2019). Clinical presentation, natural history and outcomes of intramedullary spinal cord cavernous malformations. J. Neurol. Neurosurg. Psychiatry.

[5] Santos AN, Rauschenbach L, Darkwah Oppong M (2021). Natural course of untreated spinal cord cavernous malformations: A follow-up study within the initial 5 years after diagnosis. J. Neurosurg. Spine.

[6] Moore SA, Brown RD, Christianson TJH, Flemming KD (2014). Long-term natural history of incidentally discovered cavernous malformations in a single-center cohort: Clinical article. J. Neurosurg..

[7] Kharkar S, Shuck J, Conway J, Rigamonti D (2007). The natural history of conservatively managed symptomatic intramedullary spinal cord cavernomas. Neurosurgery.

[8] Moultrie F, Horne MA, Josephson CB (2014). Outcome after surgical or conservative management of cerebral cavernous malformations. Neurology.

[9] Salman RAS, Berg MJ, Morrison L, Awad IA (2008). Hemorrhage from cavernous malformations of the brain: Definition and reporting standards. Stroke.

[10] Akers A, Al-Shahi Salman R, Awad IA (2017). Synopsis of guidelines for the clinical management of cerebral cavernous malformations: Consensus recommendations based on systematic literature review by the angioma alliance scientific advisory board clinical experts panel. Clin. Neurosurg..

[11] Arauz A, Patiño-Rodriguez HM, Chavarria-Medina M, Becerril M, Longo GM, Nathal E (2017). Rebleeding and outcome in patients with symptomatic brain stem cavernomas. Cerebrovasc. Dis..

[12] Li D, Wu Z-Y, Liu P-P (2020). Natural history of brainstem cavernous malformations: prospective hemorrhage rate and adverse factors in a consecutive prospective cohort. J. Neurosurg. JNS.

[13] Liang JT, Bao YH, Zhang HQ, Huo LR, Wang ZY, Ling F (2011). Management and prognosis of symptomatic patients with intramedullary spinal cord cavernoma: Clinical article. J Neurosurg Spine..

[14] Salman RAS, Hall JM, Horne MA (2012). Untreated clinical course of cerebral cavernous malformations: A prospective, population-based cohort study. Lancet Neurol..

[15] Chen B, Herten A, Saban D (2020). Hemorrhage from cerebral cavernous malformations: The role of associated developmental venous anomalies. Neurology.

[16] Santos AN, Rauschenbach L, Saban D (2022). Natural course of cerebral cavernous malformations in children: A five-year follow-up study. Stroke.

[17] Chen B, Saban D, Rauscher S (2021). Modifiable cardiovascular risk factors in patients with sporadic cerebral cavernous malformations: obesity matters. Stroke.

[18] Santos AN, Rauschenbach L, Darkwah Oppong M (2020). Assessment and validation of proposed classification tools for brainstem cavernous malformations. J. Neurosurg..

[19] Flemming KD, Link MJ, Christianson TJH, Brown RD (2012). Prospective hemorrhage risk of intracerebral cavernous malformations. Neurology.

[20] Flemming KD, Kumar S, Brown RD, Lanzino G (2020). Predictors of initial presentation with hemorrhage in patients with cavernous malformations. World Neurosurg..

[21] Rauscher S, Santos AN, Gull HH (2023). Modifiable vascular risk factors in patients with cerebral and spinal cavernous malformations: A complete ten-year follow-up study. Eur. J. Neurol..

[22] Dammann P, Wrede K, Zhu Y (2017). Correlation of the venous angioarchitecture of multiple cerebral cavernous malformations with familial or sporadic disease: A susceptibility-weighted imaging study with 7-Tesla MRI. J. Neurosurg..

[23] Denier C, Labauge P, Bergametti F (2006). Genotype-phenotype correlations in cerebral cavernous malformations patients. Ann. Neurol..

[24] Grambsch PM, Therneau TM (1994). Proportional hazards tests and diagnostics based on weighted residuals. Biometrika.

[25] Zhang L, Yang W, Jia W (2016). Comparison of outcome between surgical and conservative management of symptomatic spinal cord cavernous malformations. Neurosurgery.

[26] Ohnishi YI, Nakajima N, Takenaka T (2020). Conservative and surgical management of spinal cord cavernous malformations. World Neurosurg.: X.

[27] Badhiwala JH, Farrokhyar F, Alhazzani W (2014). Surgical outcomes and natural history of intramedullary spinal cord cavernous malformations: A single-center series and meta-analysis of individual patient data. J. Neurosurg. Spine.

[28] Sandalcioglu IE, Wiedemayer H, Gasser T, Asgari S, Engelhorn T, Stolke D (2003). Intramedullary spinal cord cavernous malformations: Clinical features and risk of hemorrhage. Neurosurg. Rev..

[29] Kondziella D, Brodersen P, Laursen H, Hansen K (2006). Cavernous hemangioma of the spinal cord—Conservative or operative management?. Acta Neurol. Scand..

